# Emphysematous epididymo-orchitis: imaging plays a vital part in active management: case report and literature review

**DOI:** 10.1093/jscr/rjac060

**Published:** 2022-03-09

**Authors:** Sanjana Ilangovan, Noman Ghazanfar, Sudhanshu Chitale

**Affiliations:** Barnet Hospital, Royal Free Hospital NHS Trust, London EN5 3DJ, United Kingdom; Urology Department, Whittington Hospital, London N19 5NF, United Kingdom; Urology Department, Whittington Hospital, London N19 5NF, United Kingdom

## Abstract

We hereby present a rare cause of acute scrotum secondary to *emphysematous epididymo-orchitis* (EEO). It is often not diagnosed until crepitus is palpable in the scrotal wall with spreading cellulitis, at which point it has reached an advanced stage. This case report details a 55-year-old man with poorly controlled diabetes who presented with an acute scrotum that failed to resolve with oral antibiotics in primary care. Following rapid detection of EEO on an early scrotal ultrasound scan, the patient had surgical debridement and a near-total orchidectomy with only a small stump of testis and tunica albuginea left behind. This case highlights the importance of timely imaging, particularly in diabetic men with an acute scrotum with a high clinical suspicion of intratesticular abscess. An incidental but significant finding of EEO would warrant early surgical intervention to prevent a catastrophic sequelae i.e. Fournier’s gangrene.

## INTRODUCTION

Urinary tract infections are not uncommon in middle-aged men who are immuno-compromised or have diabetes, and this may get further complicated by infection with gas-producing organisms resulting in severe epididymo-orchitis [1]. Emphysematous changes involving epididymis and testis are rare and may not be detected clinically until after gas is felt as crepitus under the scrotal skin (delayed diagnosis) or gas shadows are seen on scrotal ultrasound or computer tomography (CT) imaging (early diagnosis).

Upon surgical debridement, the appearance of gas bubbles during incision helps confirm the diagnosis. Testicular involvement with total parenchymal and tunical destruction is rather unusual.

*Escherichia coli*, originating from the lower genito-urinary tract, is one of the most common bacteria implicated in gas producing infections in diabetic patients [2]. However, with no evidence of seminal vesiculitis as a possible source and absence of genito-urinary tract fistula or recent endoscopic intervention as the two other possible underlying precipitating factors for such infection [3–6], such clinico-pathological findings of emphysematous epididymo-orchitis (EEO) are indeed rare.

Despite epididymo-orchitis being a common finding in diabetic and non-diabetic men [7], only a few cases of EEO have been reported in the literature so far [2–6, 8, 9], meaning this condition could well be under diagnosed if not under reported and perhaps missed, by the time it reached its natural outcome i.e. Fournier’s gangrene or necrotizing fasciitis.

## CASE REPORT

A 55-year-old diabetic man presented with an acute scrotum with previous history of being treated with three courses of oral antibiotics for 7 days each in the primary care and failure of resolution of his symptoms. He has poorly controlled type I diabetes. He had no history of recent endo-urological intervention, radiation, trauma or any other history to suggest any anatomical abnormalities.

Clinical examination showed grossly swollen right hemiscrotum, tender, erythematous with hard leathery skin. Digital rectal examination demonstrated non-tender benign small prostate. He was tachycardic (110 beats/min) and afebrile (37.3°C) with normal blood pressure (125/70 mmHg).

Blood chemistry showed leucocytosis (23 × 10^9^/L), raised C-reactive protein of 200 mg/L and deranged renal function: (Creatinine: 100 μmol/L, eGFR 70 ml/min/1.73 m^2^). Random blood glucose was 22 mmol/L and ketone levels were unreadable. A venous blood gas (VBG) performed revealed metabolic acidosis with lactate 2.7 mmol/L. No previous bloods or urine cultures were available.

He was started on intravenous broad-spectrum antibiotics based on advice from microbiologist. He was clinically unwell with nausea and vomiting and abdominal pain and given the raised blood glucose levels with metabolic acidosis seen on VBG, he was treated according to the diabetic ketoacidosis protocol with urgent resuscitation of intravenous fluid and insulin infusion.

**Figure 1:**
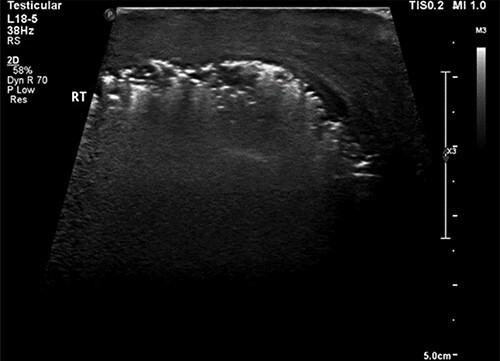
Ultrasound image of the right hemiscrotum showing gas bubbles causing acoustic shadowing and ringdown reverberation artifacts obscuring the deep tissue namely testicular parenchyma.

A scrotal ultrasound scan arranged within a few hours of presentation showed echogenic gas shadows within scrotal sac posing difficulty with visualization of underlying structures including testis and epididymis (Fig. 1). These findings were further corroborated by a pelvic non-contrast CT scan.

**Figure 2:**
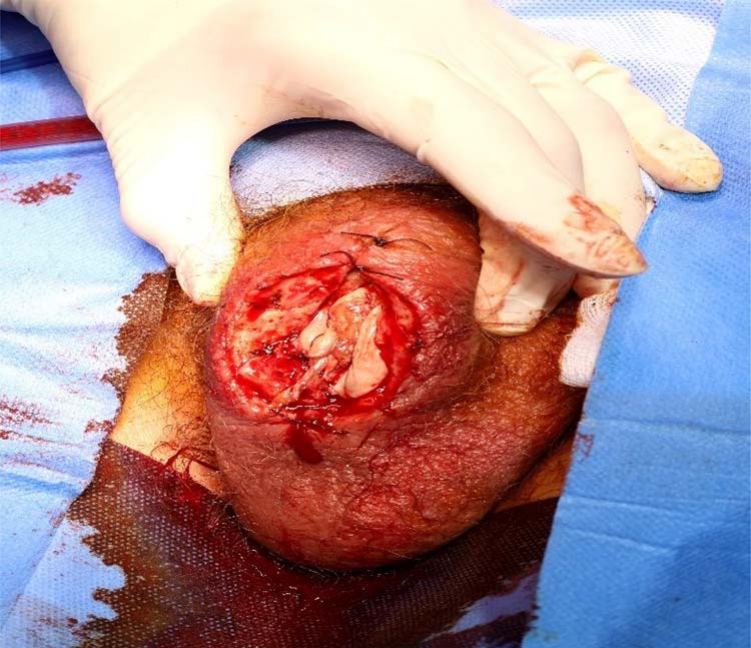
Post debridement picture showing healthy tissue with tunica albuginea stump of right testis.

Once the blood glucose level was controlled with insulin-dextrose, the patient underwent surgical debridement of scrotum under spinal anaesthesia. Appearances of diffusely scattered gas bubbles upon scrotal incision and liquefaction necrosis of lower half of testis were noted. Most of the non-viable testicular tissue was removed leaving a healthy tunical stump behind (Fig. 2).

Swabs were taken from the scrotal wound and exudate, which showed heavy growth of *E. coli*, sensitive to co-amoxiclav. Tissue cultures also showed *E. coli* sensitive to co-amoxiclav but resistant to most others.

We followed him up in clinic after 3 months. The scrotal wound had healed well and a small residual testicular stump was palpable. The residual stump was enough to fill the scrotal sac and have a psychological benefit for the patient. There was no need for prosthesis.

## DISCUSSION

Testicular involvement and near-total destruction with gas-forming bacterial infection are a rare occurrence. This is different from Fournier’s gangrene, which usually spares the testicular and epididymal tissue [10]. In most of the reported cases, it was secondary to genito-colonic fistula formation [3, 6] or recent urinary tract intervention [4].

However, even without such suggestive history, there should be a high index of suspicion for ruling out not only an intratesticular abscess (which can be managed with IV antibiotics if not surgical drainage) but the more serious complication of EEO in diabetic men who present with acute scrotum as the latter most certainly warrants surgical debridement. To establish an early diagnosis, it would be desirable to have a low threshold for organizing appropriate imaging, which is highlighted in this case, with scrotal ultrasound scan clinching the diagnosis of EEO much earlier than would be feasible on clinical assessment with palpable crepitus under the scrotal skin, which is a late development.

The wound swabs reported abundance of *E. coli* that is usually seen in emphysematous pyelonephritis [11] but rarely in EEO [7].

An early, rapid access imaging with a scrotal ultrasound is essential to establish an early diagnosis as it is paramount to implementing appropriate changes to the management and care of these patients from conservative to interventional i.e. surgical debridement.

This is in contrast with the treatment of emphysematous pyelonephritis, in which surgical intervention (nephrectomy) is reserved only for cases that completely fail to respond to conservative management and require percutaneous surgical drainage.

In conclusion, since ultrasound scanners and small part probes are easily available in most emergency radiology settings, imaging ought to be sought in selected clinical scenarios as this case clearly underpins the role of an early scrotal ultrasound scan in diabetic men with an acute scrotum to clinch an early diagnosis well ahead of clinical signs noticeable only at an advanced stage.
